# Characteristics and Clinical Outcomes of Adult Patients Hospitalized with COVID-19 — Georgia, March 2020

**DOI:** 10.15585/mmwr.mm6918e1

**Published:** 2020-05-08

**Authors:** Jeremy A. W. Gold, Karen K. Wong, Christine M. Szablewski, Priti R. Patel, John Rossow, Juliana da Silva, Pavithra Natarajan, Sapna Bamrah Morris, Robyn Neblett Fanfair, Jessica Rogers-Brown, Beau B. Bruce, Sean D. Browning, Alfonso C. Hernandez-Romieu, Nathan W. Furukawa, Mohleen Kang, Mary E. Evans, Nadine Oosmanally, Melissa Tobin-D’Angelo, Cherie Drenzek, David J. Murphy, Julie Hollberg, James M. Blum, Robert Jansen, David W. Wright, William M. Sewell, Jack D. Owens, Benjamin Lefkove, Frank W. Brown, Deron C. Burton, Timothy M. Uyeki, Stephanie R. Bialek, Brendan R. Jackson

**Affiliations:** ^1^CDC COVID-19 Emergency Response; ^2^Epidemic Intelligence Service, CDC; ^3^Georgia Department of Public Health; ^4^Oak Ridge Institute for Science and Education, Oak Ridge, Tennessee; ^5^Emory University School of Medicine, Atlanta, Georgia; ^6^Georgia Clinical & Translational Science Alliance, Atlanta, Georgia; ^7^Grady Health System, Atlanta, Georgia; ^8^Phoebe Putney Memorial Hospital, Albany, Georgia; ^9^Emory Decatur Hospital, Decatur, Georgia.

SARS-CoV-2, the novel coronavirus that causes coronavirus disease 2019 (COVID-19), was first detected in the United States during January 2020 (*1*). Since then, >980,000 cases have been reported in the United States, including >55,000 associated deaths as of April 28, 2020 (*2*). Detailed data on demographic characteristics, underlying medical conditions, and clinical outcomes for persons hospitalized with COVID-19 are needed to inform prevention strategies and community-specific intervention messages. For this report, CDC, the Georgia Department of Public Health, and eight Georgia hospitals (seven in metropolitan Atlanta and one in southern Georgia) summarized medical record–abstracted data for hospitalized adult patients with laboratory-confirmed* COVID-19 who were admitted during March 2020. Among 305 hospitalized patients with COVID-19, 61.6% were aged <65 years, 50.5% were female, and 83.2% with known race/ethnicity were non-Hispanic black (black). Over a quarter of patients (26.2%) did not have conditions thought to put them at higher risk for severe disease, including being aged ≥65 years. The proportion of hospitalized patients who were black was higher than expected based on overall hospital admissions. In an adjusted time-to-event analysis, black patients were not more likely than were nonblack patients to receive invasive mechanical ventilation^†^ (IMV) or to die during hospitalization (hazard ratio [HR] = 0.63; 95% confidence interval [CI] = 0.35–1.13). Given the overrepresentation of black patients within this hospitalized cohort, it is important for public health officials to ensure that prevention activities prioritize communities and racial/ethnic groups most affected by COVID-19. Clinicians and public officials should be aware that all adults, regardless of underlying conditions or age, are at risk for serious illness from COVID-19.

Hospitalized cases were selected to describe patients with severe manifestations of COVID-19 that warranted inpatient management. Data were collected from a convenience sample of 305 patients at seven hospitals in metropolitan Atlanta (five community hospitals, one university hospital, and one public hospital) and one community hospital in southern Georgia. Patients were selected sequentially from lists provided in real time by hospitals from a total of 698 patients aged ≥18 years who were hospitalized with laboratory-confirmed COVID-19 during March 1–March 30, 2020, including stays for observation and deaths in the emergency department. Over a 3-week period, data were abstracted from electronic medical records and recorded using Research Electronic Data Capture software (version 8.8.0; Vanderbilt University) (*3*). Hospitalizations for patients transferred between participating hospitals or admitted multiple times to the same hospital were analyzed as a single hospitalization. Data on patient race/ethnicity, age, sex, body mass index (BMI), insurance status, residence (e.g., in a long-term care facility), risk factors for severe COVID-19 (based on currently available data and clinical expertise)^§^ (*4*), and outcomes were abstracted from medical records. Race was categorized as black (non-Hispanic) or nonblack (all other racial/ethnic groups), and age was analyzed in three groups: 18–49, 50–64, and ≥65 years. Fisher’s exact tests for proportions and the Wilcoxon rank sum test or the Kruskal-Wallis H test for medians were used to test differences identified in descriptive analyses. Multivariable Cox proportional-hazards analysis was performed on the association between race and time to meeting a composite outcome of IMV or death, adjusting for age, sex, BMI, hospital, admission date, and underlying medical conditions (selected through a stepwise Akaike information criterion approach, which balances a model’s fit against its complexity); censoring was used to account for patients still hospitalized without receiving IMV. P-values <0.05 were considered statistically significant. R statistical software (version 3.6.3; The R Foundation) was used to conduct all analyses. 

Among 305 patients hospitalized with COVID-19, the median age was 60 years (range = 23–95 years, interquartile range [IQR] = 46–69 years) (Figure 1); 50.5% of patients were female, and 284 (93%) were hospitalized in metropolitan Atlanta. Data on race/ethnicity were available for 297 (97.4%) patients, among whom, 247 (83.2%) were black, 32 (10.8%) were non-Hispanic white, eight (2.7%) were non-Hispanic Asian or Pacific Islander, and 10 (3.4%) were Hispanic (Figure 2). Median age was not significantly different between black patients (60 years, IQR = 45.5–69.0 years) and nonblack patients (64.5 years, IQR = 44.8–74.0 years). Most patients had private insurance (40.1%) or Medicare (33.4%); 10.9% had Medicaid, and 14.9% were uninsured. Compared with nonblack patients, black patients were more likely to have Medicaid (13.5% versus 0.0%, p = 0.002) but not more likely to be uninsured. Overall, 20 (6.6%) patients resided in long-term care facilities before hospitalization. Current smoking was reported for 5.2% of patients.

**FIGURE 1:**
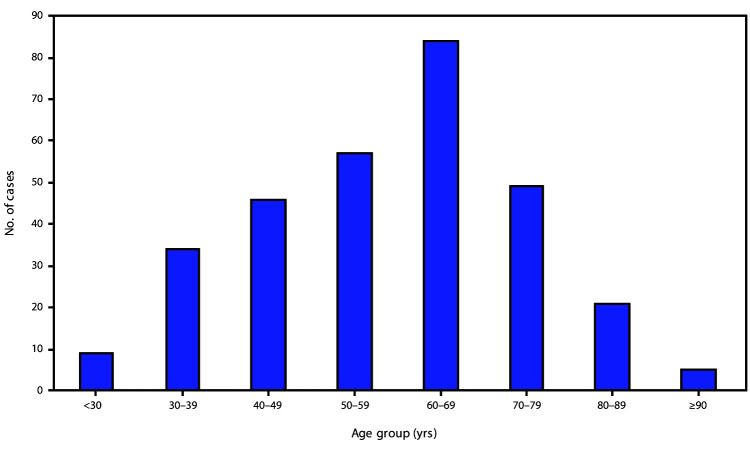
Age distribution of adults hospitalized with COVID–19 (N = 305) — eight hospitals, Georgia, March 2020 **Abbreviation:** COVID-19 = coronavirus disease 2019.

**FIGURE 2:**
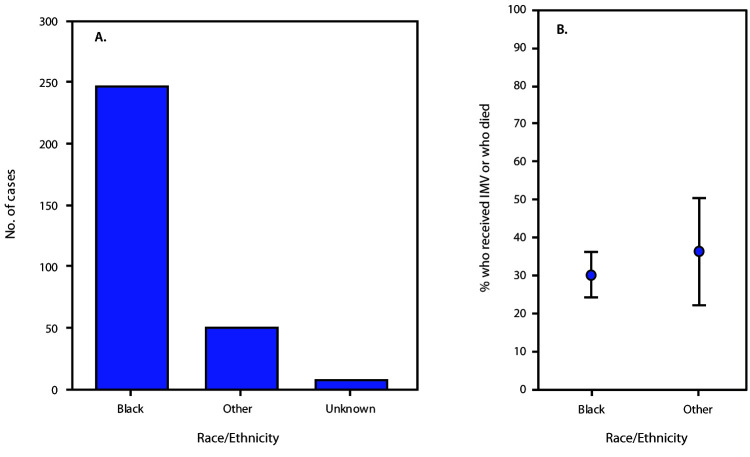
Number of hospitalized patients with COVID-19 (N = 305)* (A) and percentage who received invasive mechanical ventilation or died (B),^†^ by race/ethnicity^§^ — eight hospitals, Georgia, March 2020 **Abbreviations:** COVID-19 = coronavirus disease 2019; IMV = invasive mechanical ventilation. * A total of 273 patients had available race/ethnicity data and known hospitalization outcomes. ^†^ Vertical bars represent 95% confidence intervals for proportions. ^§^ Black was defined as non-Hispanic black race/ethnicity; other includes all other racial/ethnic groups.

Overall, 225 (73.8%) patients had conditions considered high-risk for severe COVID-19 (Table 1). Diabetes was documented in 39.7% of patients. Diabetes was most common in patients aged 50–64 years (46.5%; p = 0.001) but was not significantly more common in black patients than in nonblack patients (41.7% versus 32.0%; p = 0.21). Cardiovascular disease, documented in 25.6% of patients, was more prevalent in those aged ≥65 years (47.0%; p<0.001) but prevalence was similar in black (25.1%) and nonblack patients (30.0%) (p = 0.48). Overall, 20.3% of patients had chronic lung disease, with no significant differences by age or race. Asthma was documented in 10.5% of all patients and chronic obstructive pulmonary disease in 5.2%. Severe obesity (BMI ≥40), present in 12.7% of patients, was most common in those aged 18–49 years (21.8%; p<0.001). Severe obesity did not differ significantly by race, although median BMI was higher in black (31.4 [IQR = 27.6–36.9]) than in nonblack patients (29.6 [IQR = 24.3–32.5]; p = 0.003). Hypertension (not considered a high-risk condition) was documented in 67.5% of patients and was more common among black versus nonblack patients (69.6% versus 54.0%; p=0.047).

**TABLE 1:** Underlying medical conditions of adults hospitalized with COVID-19 (N = 305), by age group and race/ethnicity* — eight hospitals, Georgia, March 2020

Characteristic	All patients, no. (%) (N = 305)	Age group (yrs)				Race/Ethnicity*^,§^		
		No. (%)			P-value^†^	No. (%)		P-value^†^
		18–49 (n = 89)	50–64 (n = 99)	≥65 (n = 117)		Black (n = 247)	Other (n = 50)	
**High-risk conditions**								
None^¶^	80 (26.2)	47 (52.8)	33 (33.3)	N/A	0.008	62 (25.1)	16 (32.0)	0.38
Any	225 (73.8)	42 (47.2)	66 (66.7)	N/A	N/A	185 (74.9)	34 (68.0)	N/A
Diabetes mellitus	121 (39.7)	21 (23.6)	46 (46.5)	54 (46.2)	0.001	103 (41.7)	16 (32.0)	0.21
Cardiovascular disease	78 (25.6)	10 (11.2)	13 (13.1)	55 (47.0)	<0.001	62 (25.1)	15 (30.0)	0.48
Coronary artery disease	35 (11.5)	1 (1.1)	8 (8.1)	26 (22.2)	<0.001	27 (10.9)	7 (14.0)	0.63
Congestive heart failure	33 (10.8)	8 (9.0)	4 (4.0)	21 (17.9)	0.004	29 (11.7)	4 (8.0)	0.62
Arrhythmia	18 (5.9)	2 (2.2)	1 (1.0)	15 (12.8)	<0.001	11 (4.5)	7 (14.0)	0.018
Chronic lung disease	62 (20.3)	14 (15.7)	26 (26.3)	22 (18.8)	0.18	53 (21.5)	6 (12.0)	0.17
Asthma	32 (10.5)	12 (13.5)	13 (13.1)	7 (6.0)	0.12	30 (12.1)	2 (4.0)	0.13
COPD	16 (5.2)	0 (—)	7 (7.1)	9 (7.7)	0.011	14 (5.7)	1 (2.0)	0.48
Severe obesity (BMI ≥40)**	37 (12.7)	19 (21.8)	14 (14.6)	4 (3.7)	<0.001	33 (13.9)	2 (4.2)	0.088
Immunocompromising conditions or therapies^§§^	28 (9.2)	9 (10.1)	8 (8.1)	11 (9.4)	0.91	20 (8.1)	7 (14.0)	0.18
End-stage renal disease, on dialysis	16 (5.2)	4 (4.5)	5 (5.1)	7 (6.0)	0.95	15 (6.1)	1 (2.0)	0.49
Liver disease	7 (2.3)	0 (—)	4 (4.0)	3 (2.6)	0.18	4 (1.6)	2 (4.0)	0.27
**Other underlying conditions**								
No underlying conditions	18 (5.9)	13 (14.6)	1 (1.0)	4 (3.4)	<0.001	12 (4.9)	6 (12.0)	0.094
Hypertension	206 (67.5)	30 (33.7)	75 (75.8)	101 (86.3)	<0.001	172 (69.6)	27 (54.0)	0.047
Neurologic disorder	38 (12.5)	8 (9.0)	10 (10.1)	20 (17.1)	0.17	30 (12.1)	6 (12.0)	>0.99
Chronic kidney disease, without dialysis	32 (10.5)	2 (2.2)	12 (12.1)	18 (15.4)	0.003	24 (9.7)	8 (16.0)	0.21
Cancer	12 (3.9)	3 (3.4)	3 (3.0)	6 (5.1)	0.76	10 (4.0)	2 (4.0)	>0.99
Rheumatologic or autoimmune condition	8 (2.6)	1 (1.1)	5 (5.1)	2 (1.7)	0.22	6 (2.4)	2 (4.0)	0.63

**Abbreviations**: BMI = body mass index; COPD = chronic obstructive pulmonary disease; COVID-19 = coronavirus disease 2019; IQR = interquartile range; N/A = not applicable.

* Black was defined as non-Hispanic black race/ethnicity; other includes all other racial/ethnic groups.

^†^ P-values were calculated using Fisher’s exact tests for proportions.

^§^ Eight patients were excluded from race comparisons because race and ethnicity data were missing.

^¶^ Age ≥65 years was considered a high-risk condition.

** BMI data were missing for 13 patients.

^§§^ Documented conditions included solid organ transplant (eight), human immunodeficiency virus infection (eight), cancer with chemotherapy receipt within the previous year (three), stem cell transplant (three), and leukemia (two); 16 patients were taking immunosuppressive medications.

Among the 305 hospitalized patients, the median duration of hospitalization was 8.5 days and duration increased with age (Table 2). Intensive care unit (ICU) admission occurred among 119 (39.0%) patients and increased significantly with age group: among patients aged ≥65 years, 53.8% were admitted to an ICU (p<0.001). Overall, 92 (30.2%) patients received IMV, representing 77.3% of those admitted to an ICU.

**TABLE 2:** Health care use, interventions, and outcomes in adults hospitalized with COVID-19 (N = 305), by age group and race/ethnicity* — eight hospitals, Georgia, March 2020

Characteristic of hospitalization	Total no. (%) (N = 305)	Age group (yrs)				Race/Ethnicity*^,†^		
		No. (%)			P-value^§^	No. (%)		P-value^§^
		18–49 (n = 89)	50–64 (n = 99)	≥65 (n = 117)		Black (n = 247)	Other (n = 50)	
**Health care use**								
Median hospital duration, days^¶^	8.5 (5.0–14.0)	7.0 (4.3–11.8)	8.0 (5.0–12.8)	10.0 (6.0–16.0)	0.001	8.0 (5.0–13.8)	8.0 (4.0–14.0)	0.084
Any supplemental oxygen	232 (76.1)	58 (65.2)	70 (70.7)	104 (88.9)	<0.001	186 (75.3)	40 (80.0)	0.59
Nasal cannula	220 (72.1)	57 (64.0)	67 (67.7)	96 (82.1)	0.007	177 (71.7)	37 (74.0)	0.86
Noninvasive ventilation	11 (3.6)	2 (2.2)	4 (4.0)	5 (4.3)	0.80	10 (4.0)	0 (—)	0.22
High-flow nasal cannula	69 (22.6)	13 (14.6)	17 (17.2)	39 (33.3)	0.002	55 (22.3)	14 (28.0)	0.37
**ICU admission and interventions**								
Admitted to ICU	119 (39.0)	24 (27.0)	32 (32.3)	63 (53.8)	<0.001	96 (38.9)	21 (42.0)	0.75
Median ICU duration, days^¶^	8.0 (5.0–12.0)	7.0 (4.0–14.0)	8.0 (6.0–11.0)	9.0 (5.0–12.0)	0.74	8.0 (5.0–12.0)	9.0 (6.0–11.0)	0.92
Invasive mechanical ventilation	92 (30.2)	17 (19.1)	27 (27.3)	48 (41.0)	0.003	75 (30.4)	16 (32.0)	0.87
Median ventilator days^¶^	9.0 (5.0–12.0)	8.5 (5.0–13.3)	9.0 (5.5–10.5)	10.0 (6.0–12.0)	0.74	9.0 (5.0–11.5)	9.5 (6.3–13.3)	0.20
Acute renal replacement therapy	23 (7.5)	2 (2.2)	8 (8.1)	13 (11.1)	0.037	19 (7.7)	3 (6.0)	>0.99
Vasopressor support	84 (27.5)	13 (14.6)	21 (21.2)	50 (42.7)	<0.001	70 (28.3)	13 (26.0)	0.86
Cardiopulmonary resuscitation	13 (4.3)	2 (2.2)	3 (3.0)	8 (6.8)	0.25	11 (4.5)	2 (4.0)	>0.99
**Outcome**								
Discharged alive	233 (76.4)	85 (95.5)	83 (83.8)	65 (55.6)	<0.001	192 (77.7)	34 (68.0)	0.15
Still hospitalized	24 (7.9)	1 (1.1)	7 (7.1)	16 (13.7)	0.002	18 (7.3)	6 (12.0)	0.26
Died**	48 (17.1)	3 (3.4)	9 (9.8)	36 (35.6)	<0.001	37 (16.2)	10 (22.7)	0.28
Invasive mechanical ventilation or death**	86 (30.6)	16 (18.2)	22 (23.9)	48 (47.5)	<0.001	69 (30.1)	16 (36.4)	0.48

**Abbreviations**: COVID-19 = coronavirus disease 2019; ICU = intensive care unit; IQR = interquartile range.

* Black was defined as non-Hispanic black race/ethnicity; other includes all other racial/ethnic groups.

^†^ Eight patients were excluded from race comparisons because race and ethnicity data were missing.

^§^ P-values were calculated using Fisher’s exact tests for proportions and the Wilcoxon rank-sum test or the Kruskal-Wallis H test for medians.

^¶^ Continuous variables are presented as median (IQR).

** Among 281 total patients who were no longer hospitalized, 88 (31.3%) were aged 18–49 years, 92 (32.7%) were aged 50–64 years, and 101 (35.9%) were aged ≥65 years; among 273 patients with available race/ethnicity data who were no longer hospitalized, 229 (83.9%) were non-Hispanic black, and 44 (16.1) were of other race/ethnicity.

Among 281 (92.1%) patients who were no longer hospitalized at the time of data abstraction, 48 (17.1%) died. Case fatality among patients aged 18–49 years, 50–64 years, and ≥65 years was 3.4%, 9.8%, and 35.6%, respectively (p<0.001). Black patients were not more likely than were nonblack patients to receive IMV, to die, or to experience the composite outcome of IMV or death (Figure 2). Among patients without high-risk conditions, 22.5% were admitted to the ICU, 15.0% received IMV, and 5.1% died while in the hospital. As of April 24, 2020, 24 (7.9%) patients remained hospitalized, including 14 (58.3%) in the ICU and nine (37.5%) on IMV. Overall, the estimated percentage of deaths among patients who received ICU care ranged from 37.0%, assuming all remaining ICU patients survived, to 48.7%, assuming all remaining ICU patients died. In an adjusted time-to-event analysis of IMV or death as a composite outcome, no significant difference was found between black and nonblack patients (HR = 0.63; 95% CI = 0.35–1.13).

## Discussion

This report characterizing a cohort of hospitalized adults with COVID-19 in Georgia (primarily metropolitan Atlanta) found that most patients in the cohort were black, and black patients had a similar probability of receiving IMV or dying during hospitalization compared with nonblack patients. Although a larger proportion of older patients had worse outcomes (IMV or death), a considerable proportion of patients aged 18–64 years who lacked high-risk conditions received ICU-level care and died (23% and 5%, respectively). Estimated case fatality among patients who received ICU care was high (37%–49%) but comparable with that observed in a smaller case series of COVID-19 patients in the state of Washington (*5*). Among hospitalized patients, 26% lacked high-risk factors for severe COVID-19, and few patients (7%) lived in institutional settings before admission, suggesting that SARS-CoV-2 infection can cause significant morbidity in relatively young persons without severe underlying medical conditions. Community mitigation recommendations (e.g., social distancing) should be widely instituted, not only to protect older adults and those with underlying medical conditions, but also to prevent the spread of SARS-CoV-2 among persons in the general population who might not consider themselves to be at risk for severe illness (*6*).

The proportion of hospitalized patients who were black was higher than expected based on overall hospitalizations. At four affiliated hospitals, which accounted for 67% of patients in the cohort, 80% of cohort patients were black compared with 47% of hospitalized patients overall during March 2020 (D. Murphy, personal communication, April 7, 2020). Similarly, COVID-NET, which conducts population-based surveillance for laboratory-confirmed COVID-19–associated hospitalizations across 14 sites nationwide,^¶^ found that black persons were disproportionately represented among hospitalized patients with COVID-19 (*7*). It is important to continue ongoing efforts to understand why black persons are disproportionately hospitalized for COVID-19, including the role of social and economic factors (including occupational exposures) in SARS-CoV-2 acquisition risk. It is critical that public health officials ensure that prevention activities prioritize communities and racial groups most affected by COVID-19.

The findings in this report are subject to at least three limitations. First, the data are from a convenience sample of hospitalized adult patients in metropolitan Atlanta and southern Georgia, and data collection for this assessment was limited by the intention to conduct the investigation quickly. These patients do not necessarily represent all hospitalized patients with COVID-19 at those hospitals, or within Georgia. Second, patients were not tracked after discharge in this investigation. Finally, race and ethnicity were abstracted from medical records, and methods for recording these categories might have differed across hospitals, which could result in misclassification.

This report provides valuable clinical data on a large cohort of hospitalized patients. Although frequency of IMV and fatality did not differ by race, black patients were disproportionately represented among hospitalized patients, reflecting greater severity of COVID-19 among this population. Public officials should consider racial differences among patients affected by COVID-19 when planning prevention activities. Approximately one quarter of patients had no high-risk conditions, and 5% of these patients died, suggesting that all adults, regardless of underlying conditions or age, are at risk for serious COVID-19–associated illness.

**Summary**

**What is already known about this topic?**

Older adults and persons with underlying medical conditions are at higher risk for severe COVID-19. Non-Hispanic black patients are overrepresented among hospitalized U.S. COVID-19 patients.

**What is added by this report?**

In a cohort of 305 hospitalized adults with COVID-19 in Georgia (primarily metropolitan Atlanta), black patients were overrepresented, and their clinical outcomes were similar to those of nonblack patients. One in four hospitalized patients had no recognized risk factors for severe COVID-19.

**What are the implications for public health practice?**

Prevention activities should prioritize communities and racial groups most affected by severe COVID-19. Increased awareness of the risk for serious illness among all adults, regardless of underlying medical conditions or age, is needed.

## Footnotes

* COVID-19 was confirmed with laboratory detection of SARS-CoV-2 by reverse transcription-polymerase chain reaction.

† Endotracheal intubation and mechanical ventilation.

§ Persons aged ≥65 years, persons living in a nursing home or long-term care facility, persons of any age with underlying medical conditions (particularly if the condition is not well controlled), including chronic lung disease or moderate to severe asthma, serious heart conditions, immunocompromise (including cancer treatment, bone marrow or organ transplantation, immune deficiencies, poorly controlled human immunodeficiency virus infection or acquired immunodeficiency syndrome, prolonged use of corticosteroids and other immune system–weakening medications), smoking, severe obesity (body mass index ≥40 kg per m^2^), diabetes mellitus, chronic kidney disease undergoing dialysis, or liver disease.

¶ https://gis.cdc.gov/grasp/COVIDNet/COVID19_5.html.
